# What Should We Know about Drug Levels and Therapeutic Drug Monitoring during Pregnancy and Breastfeeding in Inflammatory Bowel Disease under Biologic Therapy?

**DOI:** 10.3390/jcm12237495

**Published:** 2023-12-04

**Authors:** Mathilde Barrau, Xavier Roblin, Leslie Andromaque, Aurore Rozieres, Mathias Faure, Stéphane Paul, Stéphane Nancey

**Affiliations:** 1 Department of Gastroenterology, University Hospital of Saint-Etienne, 42000 Saint-Etienne, France; mathilde.barrau@chu-st-etienne.fr; 2 CIRI—Centre International de Recherche en Infectiologie, University Lyon, Inserm U1111, University Claude Bernard Lyon 1, CNRS, UMR5308, ENS de Lyon, 69007 Lyon, France; leslie.andromaque@chu-lyon.fr (L.A.); aurore.rozieres@chu-lyon.fr (A.R.); mathias.faure@chu-lyon.fr (M.F.); stepahne.nancey@chu-lyon.fr (S.N.); 3 Department of Immunology, CIC1408, GIMAP EA3064, University Hospital of Saint-Etienne, 42055 Saint-Etienne, France; 4 Department of Gastroenterology, Lyon Sud Hospital, Hospices Civils de Lyon, University Claude Bernard Lyon 1, 69003 Lyon, France

**Keywords:** therapeutic drug monitoring, pregnancy, breastfeeding, inflammatory bowel disease

## Abstract

Data on the real long-term influences of in utero drug exposure in pregnant women on childhood development are scarce and remain not well determined and depend on the duration of in utero drug exposure and maternal drug levels. Therapeutic drug monitoring (TDM) during pregnancy may help limit fetal drug exposure while maintaining an effective dose for the treatment of the underlying inflammatory bowel disease (IBD) in women. Most antibody therapies used in patients with IBD are IgG molecules which are actively transported across the placenta, especially during the third trimester of the pregnancy. Here, we propose an up-to-date clinical review to summarize the available findings of serum drug levels in maternal blood during pregnancy, in the cord blood, infants at delivery and in breast milk of patients with IBD treated with biologics. Conversely, in comparison to adalimumab (ADA) levels, which are relatively stable during pregnancy, infliximab (IFX) drug clearance decreased significantly during the last two trimesters of the pregnancy, leading to increasing drug concentrations in the blood of the pregnant women. As most guidelines recommend using live vaccines in infants at the age of one or earlier in case of negative serum drug levels in newborns, statistical models could help clinicians in making a decision to adjust the last dose of the biologic during pregnancy and to determine the optimal date to vaccinate. Altogether, data from the literature offers strong reassurance in terms of safety for anti-TNFα therapies during pregnancy not only for IBD patients who intend to conceive, but also for pregnant women and for the physicians taking care of these patients. ADA and IFX levels in breast milk are detectable, but at very low levels, and therefore, it is recommended to pursue breast feeding under anti-TNFα therapy. Our knowledge on ustekinumab or vedolizumab levels in pregnant women remains unclear and scarce. These drugs are currently not recommended for patients with IBD in clinical practice. Therefore, TDM and proactive dose adjustment are not necessary during pregnancy since its impact on making a clinical decision have not yet been clearly demonstrated in routine practice. Overall, drug concentrations in the cord blood, an infant at birth and postpartum serum concentrations in infants, due to active placental drug transfer, may have a greater impact than the limited drug transfer in breast milk during lactation on the risk of infection and developmental outcomes. Ustekinumab and vedolizumab exposure during pregnancy and lactation are both considered low risk by the recent ECCO guidelines despite the limited data that are currently available.

## 1. Introduction

Therapeutic drug monitoring (TDM) measures specific drug levels to guide treatment changes and helps clinicians in making decisions to adjust optimal drug frequency or dose administration, subsequently improving disease outcomes. The real long-term influences of in utero drug exposure on childhood development are yet to be investigated effectively and depend on the duration of in utero drug exposure and maternal drug levels. TDM during pregnancy may theoretically help limit fetal exposure while maintaining optimal drug levels in maternal blood. However, the consequences of drug exposure in utero on immune development and maturation in childhood are critical issues and are uncertain, and the present reassuring data have reported the absence of causal relationships between adverse events (including severe infection) in neonates and maternal drug exposure with biologics. Most antibody therapies used in patients with IBD are IgG molecules known to be transported across the placenta by an active mechanism, especially during the last trimester of the pregnancy. This review proposes here a clinical review to summarize the available findings of drug levels in infants, cord and maternal during pregnancy, as well as during breastfeeding in IBD patients treated with biologics. Additionally, the usefulness of TDM in these patients during pregnancy is also be examined. In contrast with anti-TNFα agents, it is unsurprising that little data about pharmacokinetics (PK) of recently approved monoclonal antibodies (vedolizumad, ustekinumab) are available in relation to pregnancy and breastfeeding. A better understanding of biological drug pharmacokinetics during pregnancy and breastfeeding is of primary interest for gastroenterologists. This could improve dose adjustments of biological therapies in order to minimize risks of fetal exposure and to achieve an optimal maternal disease control.

### 1.1. Search Strategy

We performed a systematic review of the literature in accordance with the PRISMA guidelines of 2023. We used PubMed on 20 August 2023 to systematically search and retrieve studies on the PK of IBD-related drugs throughout the trimesters of pregnancy or in women at time of delivery and the offspring. Research was also conducted for analyzing PK in breastmilk. English articles or oral presentations during DDW, ECCO or UEGW were also analyzed. The following five main keywords including “pharmacokinetics”, “IBD related drugs”, “pregnant women”, “offspring” and “lactation” represented the search strategy. Studies including non-IBD participants were excluded. Studies which failed to meet the research aim and inclusion criteria were excluded. The search strategy and study selection were conducted by two investigators (MB and SN) independently. The results of the study selection were then discussed, and in the case of disagreement, an additional author (XR) was solicited.

### 1.2. Data Extraction

Once the relevant studies meeting the aim and inclusion criteria of this research were narrowed down, the data were extracted in a Microsoft Excel sheet. The study characteristics of interest were its design, the type of IBD, the sample size of pregnant women population enrolled in the study, the type, dosage and dosing interval of medication, the time-point of analysis (prior pregnancy (T0), first trimester (T1), second (T2), third (T3), at delivery (T4) and/or postpartum (T5)), age and bodyweight at inclusion. We also added the methods used to measure drug concentration. The timeframes for the trimesters were the following: T1 between 0–13 weeks, T2 between 14–26 weeks and T3 between 27–40 weeks. The PK parameters per study were also obtained as drug concentrations based on time between injection or infusion and drug measurements. We have no reported area under curve in the literature. Furthermore, it was also studied whether adapted dosages were advised by these studies on basis of a potential change in PK during pregnancy. The same study characteristics were analyzed in relation to lactation. For newborns, PK data were extracted according to the time of study, the type of IBD the mother is diagnosed with, the treatment and the analytical method used for measurements.

### 1.3. Mechanisms of Drug Transport for Antibody Therapies across the Human Placenta

**Figure 1:**
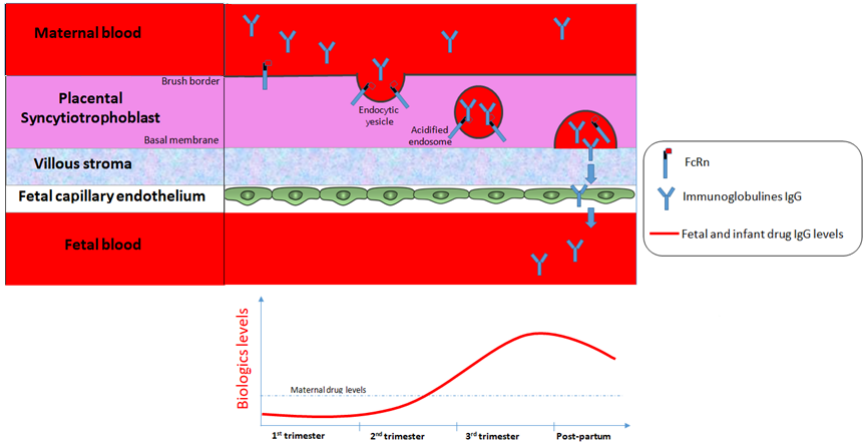
Placenta immunoglobulin G (IgG) transport.

Most drugs cross the placenta by simple diffusion of the molecules driven by concentration and electrical gradients. However, beyond simple diffusion, various other mechanisms of drug exchanges between maternal and fetal blood are involved, such as transcellular transfer (via channels, facilitated diffusion or carrier-mediated active transport, endocytosis and exocytosis) [1]. In contrast with small molecules, monoclonal antibody therapies, most of them being IgG1, are high molecular weight drugs and therefore cannot cross the placenta by simple diffusion (insignificant concentrations are detected in early pregnancy). In contrast, the maternal transfer of IgG through the placenta is mediated by active transporters using a specific receptor-mediated binding Fcγ portion of IgG at the syncytiotrophoblast layers of the placenta. These layers represent the main location of exchange for nutrients, gases or drugs between the blood of pregnant women and of the fetus. IgG is then transported across the syncytiotrophoblast layers in coated vesicles that protect them from lysosome-mediated degradation. IgG transport from mother to neonate is mediated by the heterodimer fetal Fc receptor neonatal (FcRn) molecule, including an α-chain homologous to major histocompatibility complex class I molecules and β-2-microglobulin that both play a key role in placental IgG transport, catabolism and recycling (Figure 1). Among all the subtypes of Fcγ receptors described in human placenta, the subtype III appears to contribute mostly to IgG transfer, and its expression on the surface of the syncytiotrophoblast has been detected from 13 weeks of gestation. The subsequent active transport of biologics across the placenta starts by week 13–17 and increases gradually as the pregnancy progresses, with the highest amounts of IgG being transferred from the maternal blood stream to the fetus during the third trimester [2,3,4]. At 17–22 weeks of gestation, the fetal levels of IgG represented only 5–10% of those found in maternal circulation. Acceleration of the transfer of all IgG subclasses, especially IgG1, has been reported during the third trimester. Moreover, the levels of exogenously administered IgG1 therapy in umbilical cords correlate with the timing of the last dose prior to delivery [5]. Around 26 weeks of gestation, serum fetal IgG levels reached maternal concentrations and even exceeded it by threefold (sometimes higher) at term as assessed in cord blood levels in infants [4,6].

Interestingly, the distribution of antibody therapies among the maternal, cord and fetal blood depends on multiple complex metabolic factors as well as on the maturation of the placenta [7]. The magnitude of maternal IgG transport depends on the isotype of IgG, and IgG1 is preferentially transported to the fetus in comparison to IgG4, IgG3 and IgG2, which is the least detected of all. For example, at 17–22 weeks of gestation, the fetal levels of IgG1 were reported to be threefold higher than those of IgG2. Moreover, cord blood drug levels vary depending on the type of anti-TNF agents, with a feta l/maternal ratio of 2.6 and 1.5, respectively, for IFX and for ADA. This active transfer of IgG during the second half of a pregnancy is clinically relevant since it results in a strong exposure of antibody therapies in fetus in utero and in early life period, which represents a critical period for development, maturation and programming of the immune system. In addition, these high drug levels in neonates during pregnancy could be, at least theoretically, associated with a higher susceptibility of infection.

### 1.4. Transfer of Maternal Biologics and Drugs from Breast Tissue into Breast Milk

Serum drug concentrations directly affect drug transfer from breast tissue into breast milk, and it is assumed that most drugs can be present in breast milk due to the diffusion of small chemical molecules or active transport mediated by FcRn receptors for monoclonal antibodies (a mechanism similar to the placenta). However, beyond drug levels, other factors including breast milk pH, molecule size, protein binding and breast inflammation might interfere with the transfer of drugs into breast milk. Historically, it was recommended that women receiving biologics avoid breastfeeding. However, secretory IgA represents the predominant immunoglobulin detected in breast milk, and irrespective of the biologic, drug concentrations in breast milk are very low when compared with those found in maternal serum or in the umbilical cord, and peak concentrations were seen between 24–72 h after drug administration. Although the mechanisms of intestinal absorption of immunoglobulin possibly involving FcRn remain unclear, a small fraction of ingested monoclonal antibodies by infants from breast feeding may be absorbed in the gut, as it has been well demonstrated in an infant who was not exposed to IFX during pregnancy but was exposed to the drug during breastfeeding [8]. In addition, breastfeeding while receiving biologics did not negatively interfere with the risk of infection or of fetal developmental milestones, and hence, breastfeeding in women exposed to biologics is considered to be low risk.

### 1.5. Pharmacokinetics of Anti-TNF Agents in IBD during Pregnancy

#### Study Selection

According to our study selection, 12 studies using biologics were included, nine concerning IFX, four ADA and one golimumab (GLM), and 173 participants, including 112 (70%) with Crohn’s disease (CD), 46 (29%) ulcerative colitis (UC) and 2 (1%) unspecified IBD, were enrolled.

### 1.6. Maternal Infliximab Trough Concentrations during Pregnancy

Four studies investigated serum drug trough levels in pregnant women with IBD treated with IFX. By pooling all patients (except those in the study from Bortlik M et al. [9]), all the women had detectable serum infliximab, but infliximab was detected neither in the breast milk of nursing mothers nor in the serum of breast-fed newborns in the first study analyzing this [10]. Seow et al. [11] included prospectively twenty-five pregnant women treated with IFX or ADA maintenance therapies from the University of Calgary IBD pregnancy clinic with serum bio-banking collected each trimester. Fifteen women (8 CD, 7 UC) were treated with IFX. In this cohort, the median serum trough IFX concentrations were 8.50 μg/mL (IQR: 7.23–10.07 μg/mL) during the first trimester, 10.31 μg/mL (IQR: 7.66–15.63 μg/mL) during the second trimester and 21.02 μg/mL (IQR: 16.01–26.70 μg/mL) during the last trimester. After adjusting for various parameters involved in drug clearance (albumin, body mass index and CRP levels), serum IFX trough levels increased significantly by 4.2 μg/mL per trimester during pregnancy [12]. IFX levels were measured pre-conception, in each trimester, at delivery and postpartum in maternal serum in 23 pregnant women with IBD under IFX therapy in a prospective observational study [12]. Modelling showed an increase in IFX levels of 0.16 µg/L/week (95% CI: 0.08–0.24) (*p* < 0.001), similar to the previous findings from Seow [11]. Van Eliesen et al. [13] measured placental drug transfer and exposure to IFX and etanercept in six women with autoimmune diseases (in which two had CD). Healthy term placentas were infused with 100 µg/mL IFX (n = 4) or etanercept (n = 5) for 6 h. IFX was detectable both in the cord blood and in the placenta with a cord-to-maternal ratio and a placenta-to-maternal ratio of 1.6 ± 0.4 and 0.3 ± 0.1, respectively. From experiments using ex vivo placenta drug infusion, the magnitude of drug transfer into the placenta was not different between the drugs. Fetal drug concentrations in the blood for IFX and etanercept were 0.3 ± 0.3 µg/mL and 0.2 ± 0.2 µg/mL, respectively. However, IFX drug levels were significantly superior compared to those of etanercept (19 ± 6 µg/g versus 1 ± 3 µg/g, *p* < 0.001) in the placenta. Therefore, a higher tissue drug exposure was found with IFX than with etanercept in both in vivo and in ex vivo drug-infused placentas. In line with previous studies, a retrospective study enrolling 23 pregnant IBD patients with IFX reported that drug clearance decreased significantly during the second and the last trimester, leading to an increase in maternal IFX concentration irrespective of the drug regimen [14].

Altogether, using the TDM guidance, maternal IFX drug levels may remain constant in a de-intensified regimen, despite a de-intensified drug regimen being administered to pregnant women with IBD.

### 1.7. IFX and Maternal Trough Concentration before, during and after Pregnancy

**Figure 2:**
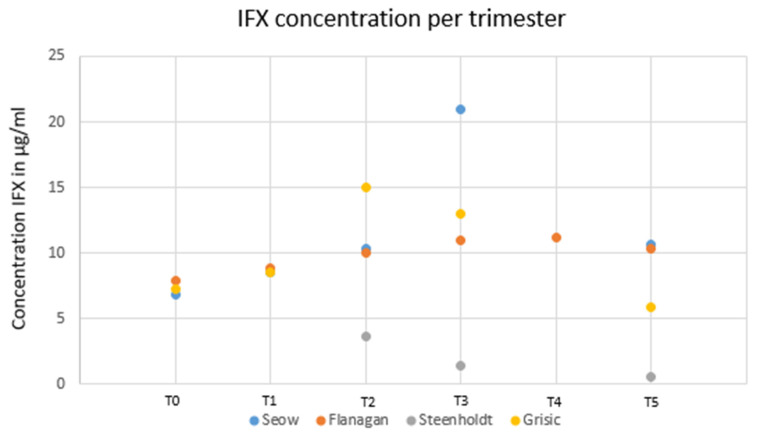
Infliximab concentration per trimester from all available studies.

Seow et al. and Flanagan et al. have showed that drug levels after delivery were higher compared to those during the pre-pregnancy period (10.17 µg/mL versus 6.9 µg/mL and 10.3 versus 7.9 µg/mL, respectively, in contrast with data from the study of Grišic et al.) (5.9 µg/mL versus 7.3 µg/mL) [11,12,14]. Overall, when comparing the IFX levels during and after pregnancy, they were all higher during pregnancy. Figure 2 reports the dynamics of drug levels before, during and after pregnancy.

### 1.8. Placenta Drug Transfer in Pregnant Women Treated with Infliximab

Two studies have reported some small case reports investigating drug levels in the blood of breast-fed infants from mothers exposed to IFX. In the first case, the breast-fed infant’s serum IFX level was 39.5 microg/mL at 6 weeks after birth [15]. In this case, a last infusion of IFX (10 mg/kg) was administered to the mother two weeks before delivery. In another study, a woman with UC receiving a common dose of IFX infusions until gestation week 31 gave birth to a healthy child at gestation week 37 [16]. Relatively high maternal serum drug levels were reported during pregnancy. In addition, detectable IFX drug levels were found in the infant’s blood at week 16 after birth, but not at reassessment at week 28. In 11 IBD pregnant patients treated with IFX, drug concentrations in the cord blood and in the blood of an infant at birth were compared with those of the mother [15]. Not only was IFX detectable in the blood of the infants for as long as 6 months, but also the median level of IFX in the cord was 160% higher compared with that of the mother. In a study including 32 CD pregnant patients treated with IFX, there was a relationship between IFX cord levels and the gestational week of last exposure as well as maternal serum levels [9]. In fact, anti-TNF drug levels in the cord blood at birth depend on the type of anti-TNF type. In a recent prospective single-center study, including 131 pregnancies that resulted in a live birth in women with IBD treated with IFX (n = 52) and ADA (n = 58). At birth, drug levels in the 94 cord blood samples were significantly higher for women treated with IFX than those treated with ADA. Interestingly, whereas the transport of ADA across the placenta was relatively limited and increased in a linear fashion during the third trimester, IFX transportation increased exponentially [17].

### 1.9. IFX Drug Levels during Breastfeeding

In a large prospective multicenter study analyzing the drug concentrations in 72 breast milk samples from patients treated with IFX therapy, drug was detected in breast milk in 19 out of 29 exposed women (with a maximum drug concentration of 0.74 μg/mL) [18].

### 1.10. Duration of IFX Detection in Newborns

In a prospective multicentric study involving 44 pregnant women treated with IFX, the authors investigated the drug concentrations in cord blood of newborns and drug clearance after birth, and the relationships between these factors and IFX levels in mothers at birth and the subsequent risk of infection in infants during the first year of life. The time from the last exposure to IFX during pregnancy was found inversely associated with drug levels in the umbilical cord (IFX: r = −0.77, *p* < 0.0001) and in the blood of women at time of birth (IFX, r = −0.80; *p* < 0.0001 for both). The median ratio of infant/mother drug concentration at birth was 1.97 (95% CI, 1.50–2.43). The mean drug clearance time in infants was 7.3 months (95% CI, 6.2–8) In this study, 4 (5%) and 16 (20%) infants experienced bacterial infections and non-serious viral infections, respectively. Infants whose mothers received a combination of an anti-TNF agent and thiopurine had a 2.7-fold higher risk of infection compared with those treated with an anti-TNF monotherapy (95% CI, 1.09–6.78; *p* = 0.02). Drugs were not detected in infant blood after the age of 12 months [5]. In a prospective study including 107 infants exposed to anti-TNF during pregnancy (in which 66 were under IFX), the authors proposed a pharmacokinetic model to predict time for drug clearance after birth in infants exposed during pregnancy. All infants with detectable drug levels in cord blood at birth and with at least one additional blood sample within the first year were enrolled. Drugs were detectable in the blood of 25 infants (23%) at 6 months. At 12 months, IFX was detected in three infants (4%) whereas ADA was undetectable. Using a Bayesian forecasting method based on a one-compartment PK model, the predicted drug clearing time was related with the measured observations [19]. In a recent study [20], the authors proposed a physiologically based pharmacokinetic (PBPK) model for anti-TNF therapies in adults and extrapolated the results to pregnant women, fetuses and infants with the objective to identify the best timing for the last dosing of IFX, ADA and GLM during pregnancy in IBD, and with the objective to study the recommended vaccine schedules for infants exposed to these drugs. The main results are reported in Table 1 and Table 2 and are of interest for clinical practice. The timing of the last dosing of IFX and ADA was defined by the lowest limit of the therapeutic range. Optimal IFX trough concentrations were considered between 3–7 μg/mL [21]. However, IFX trough concentrations over 15 μg/mL increase the likelihood of infection [22]. For pregnant women exposed to ADA, numerous studies failed to demonstrate a link between ADA trough concentrations and the increased risks of infection. Hence, to adjust optimal drug level, taking blood samples throughout pregnancy in women with IBD is recommended, as shown by Mahadevan et al. [23].

### 1.11. TNF-α Inhibitors—ADA and Maternal Trough Concentration during Pregnancy

**Figure 3:**
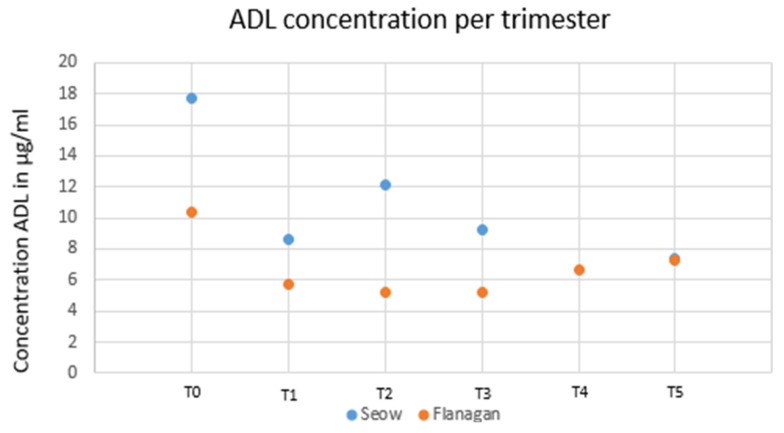
Adalimumab concentrations per trimester from two available studies.

Two studies analyzed adalimumab serum levels during pregnancy in IBD patients. Seow et al. analyzed 11 pregnant patients with IBD treated with ADA. After adjusting for albumin, BMI and CRP, drug levels remained stable (*p* > 0.05) during pregnancy [11]. Flanagan et al. [12] included 15 IBD patients treated with IFX (n = 23) and with ADA (n = 15) and with vedolizumab (n = 12) with at least two intrapartum observations. Conversely, when compared to IFX, modelling showed no change in ADA levels. These results are reported in Figure 3.

### 1.12. Placental Transfer of ADA

Two studies analyzed placental transfer of adalimumab. Mahadevan et al. [6] investigated it in 10 pregnant women with IBD treated with ADA. Serum drug levels were compared at birth in the mother, infant and cord blood, and then monthly in the infant until the drugs were undetectable. The median level of ADA in the cord was 153% than that measured in blood of the mother. In addition, the drug remained detectable in the infants for as long as 6 months. Borthlik et al. [9] analyzed the correlation between serum anti-TNF-α concentrations in the blood of infants and mothers at delivery with gestational age at the last exposure. Conversely, when compared to IFX, no correlation was found in the case of ADA for this.

### 1.13. ADA and Breast Milk (Table 3)

In a large and prospective multicenter study, among the 72 breast milk samples, ADA was detected in 2 of the 21 women under treatment with a maximal drug concentration of 0.71 μg/mL [18]. As with IFX, the maternal use of ADA appears to be compatible with breastfeeding.

**Table 3:** Breast milk drug levels.

Drug	Total Patients	Total Patients with a Detectable Level, n (%)	Peak Time Range, h	Peak (Range), μg/mL
Adalimumab	21	2 (9.5)	12–24	0.71 (0.45–0.71)
Infliximab	29	19 (66.0)	24–48	0.74 (0.15–0.74)
Golimumab	1	0 (0)	NA	NA
Certolizumab	13	3 (23.0)	24–48	0.29 (0.27–0.29)
Ustekinumab	6	4 (66.7)	12–24	1.57 (0.72–1.57)
Natalizumab	2	1 (50.0)	24	0.46

NA—Not applicable.

### 1.14. Duration of ADA in Newborns

Through a large and prospective multicenter study including 44 pregnant women treated with ADA, the authors investigated the concentrations of IFX in umbilical cord blood of newborns and the rates of drug clearance after birth. They also analyzed the relationship between drug concentrations in mothers at birth and the risk of infection during the infant’s first year of life [5]. There was a negative relationship between time from last exposure to ADA during pregnancy and drug concentration in the umbilical cord (r = −0.64, *p* = 0.0003) and in mothers at time of birth (ADA, r = −0.80; *p* < 0.0001). The median ratio of infant/mother drug concentration at birth was 1.21 for ADA (95% confidence interval (CI), 0.94–1.49), whereas the mean time of ADA clearance in infants was 4.0 months (95% CI, 2.9–5.0), and contrarily, IFX was cleared slower than ADA.

### 1.15. TNF-α Inhibitors—GLM Maternal Trough Concentration during Pregnancy, in Breast Milk and in Children

Very little data about maternal drug trough concentration are available with GLM therapy during pregnancy. Only one case study [24] covered GLM, reporting data exclusively at delivery (6.6 mcg/mL). For GLM, no advice on dosage was provided by the authors. Currently, no data are published about the evolution of serum levels of GLM during pregnancy, and thus, we do not know if the pharmacokinetics are similar to that of IFX or ADA.

Data on the use of GLM therapy during breastfeeding remain scarce (Table 3). Given the high molecular weight of GLM (around 150,000 Da), it is likely that a very low amount of drug is detected in milk samples during breastfeeding. In addition to low GLM concentration in milk, it is also likely to be partially degraded during digestion, leading to a very low drug exposure to the breastfed infant. However, we need more information on this topic, and in the meanwhile, GLM should be used with caution during breastfeeding, especially if nursing a newborn or preterm infant. Drug transfer to an infant may be minimized by waiting for at least 2 weeks postpartum. Matro et al. [18] have reported the case of one mother treated with GLM, and they failed to detect GLM in breast milk samples.

No publication is available about the exposure duration of GLM in children from mothers using GLM during pregnancy. Based on very little data reporting very low drug concentration in children post-birth, we can speculate a similar management of live vaccines in newborns as with the other anti-TNF drugs.

### 1.16. Outcomes of Pregnancy and Children When Using Anti-TNF during Pregnancy

In a recent meta-analysis pooling wight studies with a total of 527 pregnant women with IBD. A total of 343 were treated with IFX and 184 with ADA [25]. Compared to ADA, adverse pregnancy outcomes including congenital malformations and spontaneous abortion were not increased in case of exposure to IFX. Another meta-analysis has reported adverse pregnancy outcomes (APOs), congenital abnormalities (CAs), preterm birth (PTB) and low birth weight (LBW) to assess the risks associated with anti-TNFα therapy for pregnancy outcomes [26]. Anti-TNFα agents were not associated with an increased risk of APOs, CAs, PTB or LBW in comparison with disease-matched controls. Moreover, when comparing with the risk of CAs in the general population, there was no increased risk under anti-TNF therapy. Altogether, these data provide some reassurance for IBD patients and clinicians in terms of safety profile of anti-TNFα therapy during pregnancy. Finally, a separate recent meta-analysis [27] included 48 studies to assess the prevalence of adverse pregnancy outcomes in women with IBD exposed to biologic therapy. They failed to detect any difference in terms of adverse pregnancy outcomes amongst pregnant women with IBD exposed to biological therapy compared with that of the general population. In all studies, TDM was not analyzed to identify an association between serum drug levels and adverse events.

Moreover, some studies analyzed the impact of monoclonal antibody therapy use during pregnancy and the response to vaccination in newborns. In a large study including 179 women exposed to biologics, when measuring antibody titers after vaccination against HiB and tetanus toxin, the vaccine efficacy in their infants of at least 7 months of age did not appear to be affected by in utero drug exposure [28]. Julssgaard et al. [5] analyzed the concentrations of anti-TNFα in mothers and newborns and reported the risk of infections during the time. They reported bacterial and viral infections in 4 (5%) and 16 (20%) infants, respectively, and all were infectious events with benign courses. They estimated the relative risk for infection to 2.7 in infants whose mothers were treated with a combotherapy (anti-TNF agent and thiopurine), compared with anti-TNF monotherapy (95% CI, 1.09–6.78; *p* = 0.02). A prospective cohort study [29] including 191 children (IFX (67 [35%] of 191) and ADA (49 [26%])) investigated whether live rotavirus vaccine could be administered safely to infants exposed to biologic agents. They did not report severe adverse events after immunization except for three (2%) infants requiring medical attention. So, according to the authors, rotavirus vaccination is safe and can be proposed to infants exposed to anti-TNF agents in utero. A systematic bibliographic search was performed recently to assess the effectiveness and safety of vaccines in children exposed to biological drugs in utero and/or those whose mothers received biological agents during lactation [30]. Vaccines were considered to be effective in infants exposed to anti-TNF agents in utero. In contrast to live-attenuated vaccines which should be avoided while drug levels are detectable in the infants, inactivated vaccines are likely safe. All vaccines (including live-attenuated and inactivated) are possibly safe in children breastfed by mothers treated with anti-TNF therapy. However, drug levels during pregnancy or in cord blood were not concomitantly measured in this study in order to look for an association with the very low drug concentrations found in breast milk.

When can we resume IFX and ADA during pregnancy?

Firstly, studies have reported the increased levels of IFX concentrations during the third trimester of pregnancy, contrary to ADA. So, using TDM, we can speculate the possibility of decreasing the dose of IFX during this time to obtain therapeutic concentrations. The IFX concentrations in Figure 2 report discrepancies at T5 for two studies [10,15]. These discordances at T5 may possibly be explained by various time-points of drug measurement after delivery. Kane et al. and Vasilauskas et al. provided drug measurements at 14 weeks, whereas Seow et al. [11] and Flanagan et al. [12] considered post-pregnancy measurements up to 6 months [14,15]. Grisic et al. [14] reported drug assessments up to 250 weeks after conception, and Steenholdt et al. [16] proposed their last measurement at 28 weeks after delivery. However, we think that it would be interesting to measure TDM of IFX and ADA after delivery, using a proactive strategy.

For breast milk, anti-TNF concentrations are very low, and breast feeding during IFX or ADA administration is recommended to be safe. The more important point is about serum levels in newborns. Mahadevan et al. suggested performing biologic therapy for weeks before delivery. Indeed, no effects in pregnant women and newborns were reported in all studies, except for serum anti-TNF concentrations in newborns. Finally, the important question is to identify the best timing for the last dosage of IFX, ADA and GLM in pregnant women with IBD, as well as to propose the recommended vaccine schedules for infants exposed to these drugs. A PK model to predict time-to-clearance in infants exposed to anti-TNF agents during pregnancy has been developed by Liu et al. [19]. According to their online results, they can predict the duration of anti-TNF in the child and can adapt the date of live vaccination [31]. Chen et al. [20] recommend more stringent points for timing the last dose and vaccine according to the regimen used for pregnant women. However, for practitioners not using TDM, it would be easier to time live vaccines at an age of one year for the child or to discuss vaccination before the completion of one year in case no drug is detected in blood.

### 1.17. Serum Ustekinumab Levels during Pregnancy and Breastfeeding (Figure 4)

Ustekinumab is an entirely humanized IgG1 monoclonal antibody blocking the p40 subunit of interleukin (IL) IL-12 and IL-23 and is currently approved for the treatment of CD and UC. Interleukin-12 targeted by ustekinumab contributes to uterine physiology (uterine angiogenesis), the regulation of trophoblast invasion and local vascular remodeling during implantation of an embryo [32]. Notably, both high and low levels of IL-12 in pregnancy have been associated with early spontaneous abortions. In addition, IL-23 regulates the critical functions of human decidual immune cells, which play a key role in the tolerance of genetically different (allogenic) cells while aiding the mother’s immune function in early pregnancy [33]. These findings raise the concern of the potential interference of ustekinumab exposure on pregnancy. However, clinical results from a large registry comparing the pregnancy events occurring in IBD patients exposed and not exposed to ustekinumab are reassuring and did not show any unexpected adverse outcomes on pregnancy and child growth. Data in the literature on ustekinumab PK during pregnancy remain unclear, limited to small case series, and concern exclusively drug measurement in maternal, cord and infant blood and not drug concentrations in intestinal tissues. Sako M et al. investigated drug concentrations at delivery in maternal peripheral and cord blood from a patient with CD treated with ustekinumab and in infants’ blood at six months [34]. Similar to anti-TNF agents, the level of ustekinumab in cord blood was 2.8-fold higher compared with that measured in maternal serum, but drug concentration was undetectable in the baby’s bloodstream after six months [34,35,36]. Similar findings were reported in three other case reports, one in a pregnant women with CD treated until 33 weeks of gestation with the dose interval of ustekinumab shortened to every 4 weeks, another in a woman with refractory CD treated with ustekinumab until week 30 of pregnancy [35], and finally, in a patient with UC under usual maintenance therapy with a 2–2.5 times higher drug concentration in cord blood than in contemporaneous maternal serum. In the last case, the drug was still detectable in infant serum more than 2 months after the last maternal dose [37]. Interestingly, maternal drug serum trough levels which were monitored at induction therapy and throughout pregnancy were found to be stable [38].

**Figure 4:**
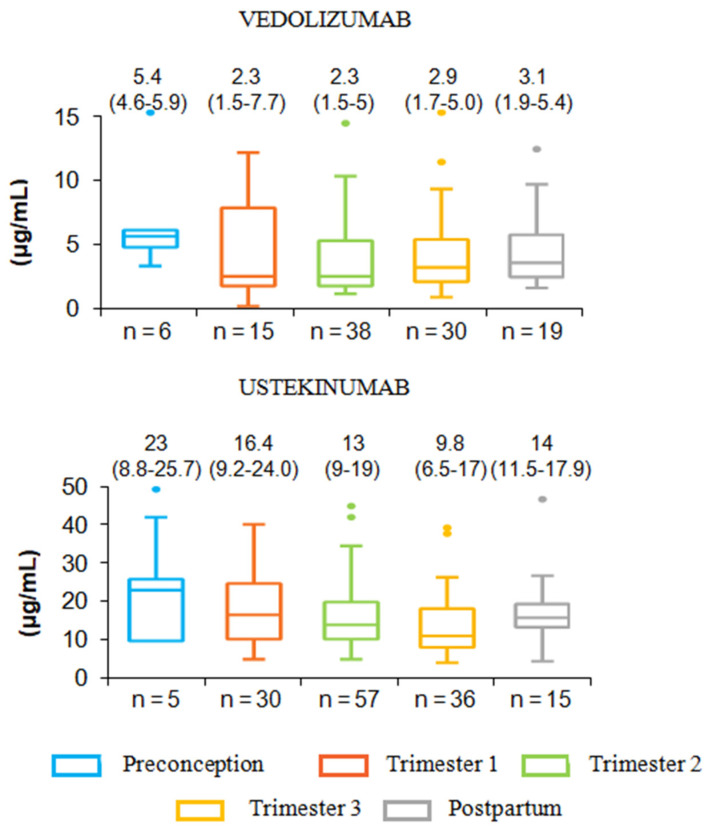
Pharmacokinetics of pregnant women with IBD.

Recently, several larger prospective observational cohort studies investigated the pharmacokinetics of ustekinumab during pregnancy. The first one from Mitrova K et al. reported drug levels in 15 infant–mother pairs during pregnancy in IBD patients [39]. At the time of delivery, drug levels in the cord and in the maternal blood were strongly correlated with a median infant-to-maternal ratio of 1.7, concordant with those previously observed in the precedent small case series. The second study presented during the last ECCO Congress which is not yet published reported the Australian experience of PK of ustekinumab and vedolizumab in pregnant women with IBD. This multicenter prospective cohort study included 35 pregnant patients treated with the maintenance dose of ustekinumab. They investigated both infant and maternal drug levels at delivery, as well as drug concentrations in infants at various time-points until drug clearance. They reported a strong positive correlation between drug levels in maternal and infant sera at time of delivery, and the infant/maternal drug level ratio was 1.74 (IQR 1.24–3.5) in accordance with that previously reported in the previous case series [40]. Time to drug clearance in infants was 13 weeks (IQR 9–22), and serum ustekinumab was not detected 15 weeks after delivery in two-third of infants. Interestingly, in this cohort, time to clearance in infants did not differ between women who received the last dose of ustekinumab during the second and those that received it during the third trimester of pregnancy. The third prospective study included 15 women with CD under ustekinumab treatment and confirmed previous data with infant drug levels at delivery that were 1.79 (IQR 1.26–3.1) fold higher than maternal levels with a median clearance time of 9 weeks (range 6–19) in nine infants in whom drugs levels were measured over time [36]. In the Pregnancy in Inflammatory Bowel Disease and Neonatal Outcomes, PIANO registry, a prospective observational study that included pregnant women with IBD in 30 US centers, similar pharmacokinetics findings of ustekinumab during pregnancy were observed in a small subgroup of patients [6].

Altogether, few studies determined ustekinumab PK during pregnancy; however, their results are in line with the overall stable concentrations of maternal serum drug levels throughout pregnancy and in infants. A lack of correlation was seen between ustekinumab concentrations in the umbilical cord blood with the gestational week during which the drug was administered. In addition, drug clearance time was not different regardless of the time of the last ustekinumab injection during the second half of pregnancy.

### 1.18. Ustekinumab Levels during Breastfeeding (Table 3)

Limited data on breast milk transfer of ustekinumab from women to their infants are available. Drug exposure of infants to ustekinumab during breastfeeding and whether drug exposure in infants has an impact on the risk of infection and developmental milestones remains unclear and controversial. A recent case report of a 36-year-old female with UC treated with a maintenance regimen of ustekinumab (90 mg/8 weeks) has provided information on drug concentrations in breast milk which were substantially lower compared with those found in cord blood (ratio 1 to 100) or in maternal serum (ration 1 to 20) and decreased gradually thereafter. When comparing serum maternal ustekinumab levels with those in breast milk, the ratio of area under the time–concentration curves of the drug was very low (0.0008) [37]. Ustekinumab was also detected in low concentration in breast milk from a small case report including three nursing mothers with CD exposed to treatment (90 mg/8 weeks for two of them and intensified dose of 90 mg/4 weeks for the latter) [41].

Notably, the low drug concentrations measured in breast milk samples collected one hour after the completion of drug administration were two-fold lower compared with pre-dose serum levels. A multicenter prospective observational study investigated breast milk samples from six women with IBD treated with ustekinumab. The low levels of ustekinumab in breast milk were detected in four out of six patients with a peak concentration of 1.57 μg/mL (ranging from 0.72–1.57). This was seen 12–72 h post-injection and was followed by a gradual decrease thereafter. In addition, infection rates and developmental delay at 12 months did not differ between drug-exposed and non-exposed breastfed infants [18].

Altogether, our knowledge on the usefulness of monitoring serum ustekinumab or vedolizumab maternal levels during pregnancy remains unclear and is currently not recommended in patients with IBD. TDM during pregnancy and proactive dose adjustment are not necessary since their impact on making clinical decisions has not been demonstrated in routine clinical practice. Overall, drug concentrations at birth in blood infant cord and in serum after birth, due to placental drug transfer, may have a greater impact on the risk of infection and development outcomes than drug transfer during lactation.

### 1.19. Serum Vedolizumab Trough Levels during Pregnancy (Figure 4)

Vedolizumab (a humanized monoclonal antibody antagonizing α4β7 integrin receptors) was approved for the treatment of IBD patients. The receptor targeted by vedolizumab inhibits leukocyte trafficking into the gut and subsequently reduces the recruitment of immune and inflammatory cells. It is also involved in placental development which has caused apprehension about its use in pregnant women. Data about the potential impact of vedolizumab exposure during pregnancy are limited and are mainly results from the European retrospective multicenter case–control observational CONCEIVE study. The study reports pregnancy and child developmental outcomes in 73 women with IBD exposed to vedolizumab. Additionally, a retrospective cohort study from the GETAID including 44 drug-exposed women with IBD during pregnancy and a few cases from the PIANO registry also contribute to the available data [42,43]. There was no clear evidence of a negative safety signal although further larger, prospective and dedicated studies are required to confirm these reassuring findings.

At the time of delivery, vedolizumab was detectable in the serum of infants. Although the mechanisms of transfer of vedolizumab across the placenta to the fetal blood stream are similar to the IgG antibodies, placental pharmacokinetic studies have reported substantial differences between vedolizumab and other biologics including anti-TNF agents and ustekinumab. A comparative study investigating the placental pharmacokinetics of vedolizumab and ustekinumab in women with IBD (15 exposed to ustekinumab and 16 to vedolizumab) found lower drug concentrations of vedolizumab in the cord blood than in the maternal blood. This is in contrast with concentrations measured in mothers exposed to ustekinumab during pregnancy. In this cohort, the median vedolizumab concentrations in maternal and in cord blood samples from mothers with IBD was 7.3 mg/L and 4.5 mg/L with a median infant-to-maternal ratio of 0.66 (compared with 1.7 in the cohort of pregnant mothers exposed to ustekinumab) [39]. Moreover, there was a positive relationship between drug levels in the cord blood and the gestational week of the last vedolizumab infusion.

### 1.20. Serum Vedolizumab Trough Levels during Breastfeeding (Table 3)

There are limited data regarding the impact of vedolizumab exposure in women with IBD during breastfeeding on infant outcomes. All data available during pregnancy and breastfeeding concern intravenous vedolizumab administration, and no data are available with the recently approved subcutaneous injection of vedolizumab.

Vedolizumab is detected in breast milk at low concentrations. Drug levels in breast milk from five vedolizumab-treated lactating women with IBD were investigated in a short report. Breast milk samples were collected prior to infusion, 30 min post-infusion and then twice daily for up to 14 days. Vedolizumab was detected in all breast milk samples with varying concentrations which were very low based on the corresponding serum concentration (less than 1%). The peak drug concentration was 0.318 μg/mL in this cohort and was raised from 3–7 days after infusion. Taking into account the maximal vedolizumab concentrations in milk samples and the overall amount of milk ingested by the infants (around 150 mL per kilogram of body weight), it is estimated that the infants could receive 0.048 mg per kilogram body weight per day [5].

Altogether, we should keep in mind that vedolizumab is detected in maternal and cord blood, and drug concentrations in the cord blood are lower than in maternal blood in the majority of cases, in contrast to those measured for ustekinumab. Safety data on the use of vedolizumab during pregnancy and breastfeeding do not indicate any negative effects. Ustekinumab and vedolizumab exposure during pregnancy and lactation are both considered low risk by the recent ECCO guidelines despite the limited available data [44]. However, we require more prospective, real-life and larger dedicated studies to confirm these reassuring findings.

## 2. Conclusions

It is actually well established among clinicians to resume the treatment of pregnant women with IBD. The benefit/risk ratio of the drug is evaluated, taking into account both the mother and the fetus. It is of great interest to maintain IBD control in remission and to minimize the subsequent risks on pregnancy outcomes, such as miscarriages and pre-term birth. Recent guidelines of the European Crohn’s and Colitis Organization (ECCO) as well as recommendations from the American Gastroenterological Association (AGA) are available and both state that maintaining IBD in remission is central to reduce adverse outcomes. Both consider biologic therapy to be safe when used for maintenance regimen during pregnancy. However, there is no clear recommendation on the use of TDM during pregnancy or in the management of newborns from mothers exposed to biologics during pregnancy. However, in contrast to other biologics, given the reduced IFX clearance during pregnancy, the use of TDM in pregnant mothers treated with IFX might help clinicians to adjust the therapeutic dose of IFX during this period. During lactation, low drug concentrations are detected in breast milk, and subsequently, breast feeding can be considered safe with the administration of anti-TNF agents. Limited data on breast milk transfer of ustekinumab, vedolizumab and golimumab from women to their infants are available. However, preliminary results are safe with very low levels of the drug. So, for anti-TNF drugs, breast feeding can be considered safe with the administration of these biologics. The essential question is to determine the best timing for the last dosing of all anti-TNF agents available for pregnant women with IBD, as well as to assess the recommended vaccine schedules for infants exposed to these drugs. Using models, the duration of anti-TNF drug persistence in a child post-birth can be predicted, allowing the identification of the optimal date to inject life vaccines in infants in a personalized manner. However, for physicians not using TDM in a daily practice, it should be easier to time live vaccines at an age of one year for the child or to discuss vaccination before the completion of one year in the absence of drug detection in the blood.

## Figures and Tables

**Table 1:** Recommended timing of last dosing.

Drug	Dose and Frequency	Timing of Last Dose (Weeks before Delivery)
Adalimumab	40 mg/2 W	0–2
	40 mg/W	0–3
	80 mg/2 W	0–4
Infliximab	5 mg/kg/8 W	5–11
	5 mg/kg/6 W	6–13
	7.5 mg/kg/8 W	8–13
	8 mg/kg/8 W	8–13

**Table 2:** Recommended timing of vaccine.

Drug	Dose and Frequency	Timing of Vaccination (Months after Delivery)
Adalimumab	40 mg/2 W	8
	40 mg/W	9
	80 mg/2 W	9
Infliximab	5 mg/kg/8 W	11
	5 mg/kg/6 W	12
	7.5 mg/kg/8 W	12
	8 mg/kg/8 W	12

## Data Availability

Data in this review of articles are contained within the article.
